# A Mediterranean-like fat blend protects against the development of severe colitis in the mucin-2 deficient murine model

**DOI:** 10.1080/19490976.2022.2055441

**Published:** 2022-04-26

**Authors:** Natasha Haskey, Jiayu Ye, Mehrbod Estaki, Andrea A. Verdugo Meza, Jacqueline A. Barnett, Mitra Yousefi, Blake W. Birnie, Samantha Gruenheid, Sanjoy Ghosh, Deanna L. Gibson

**Affiliations:** a Department of Biology, University of British Columbia - Okanagan Campus; Kelowna, British Columbia, Canada; b Department of Pediatrics, University of California, San Diego, La Jolla, California, USA; c The Center for Phenogenomics Infection & McGill University Research Centre on Complex Traits; McGill University, Montreal, Quebec, Canada; d Department of Medicine, University of British Columbia - Okanagan Campus, Kelowna, British Columbia, Canada; e Associate Professor - Department of Microbiology and Immunology, McGill University, Montreal, Quebec, Canada

**Keywords:** Colitis, ulcerative colitis, inflammation, dietary fat, mediterranean diet, mucin 2, diet, nutrition, bacteriome

## Abstract

There is a growing appreciation that the interaction between diet, the gut microbiota and the immune system contribute to the development and progression of inflammatory bowel disease (IBD). A mounting body of scientific evidence suggests that high-fat diets exacerbate IBD; however, there is a lack of information on how specific types of fat impact colitis. The Mediterranean diet (MD) is considered a health-promoting diet containing approximately 40% total fat. It is not known if the blend of fats found in the MD contributes to its beneficial protective effects. Mice deficient in the mucin 2 gene (Muc 2^−/−^) were weaned to 40% fat, isocaloric, isonitrogenous diets. We compared the MD fat blend (high monounsaturated, 2:1 n-6:n-3 polyunsaturated and moderate saturated fat) to diets composed of corn oil (CO, n-6 polyunsaturated-rich), olive oil (monounsaturated-rich) or milk fat (MF, saturated-rich) on spontaneous colitis development in Muc2^−/−^ mice. The MD resulted in lower clinical and histopathological scores and induced tolerogenic CD103+ CD11b+ dendritic, Th22 and IL-17+ IL-22+ cells necessary for intestinal barrier repair. The MD was associated with beneficial microbes and associated with higher cecal acetic acid levels negatively correlated with colitogenic microbes like *Akkermansia muciniphila*. In contrast, CO showed a higher prevalence of mucin-degraders including *A. muciniphila* and Enterobacteriaceae, which have been associated with colitis. A dietary blend of fats mimicking the MD, reduces disease activity, inflammation-related biomarkers and improves metabolic parameters in the Muc2^−/−^ mouse model. Our findings suggest that the MD fat blend could be incorporated into a maintenance diet for colitis.

## Graphical abstract

**Figure:**
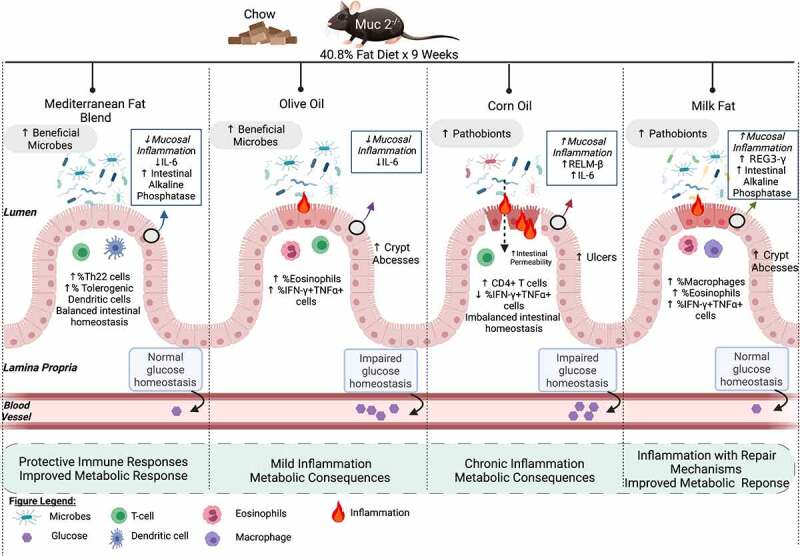

## Introduction

Inflammatory bowel diseases (IBD), including both ulcerative colitis (UC) and Crohn’s disease (CD), are characterized by chronic intestinal inflammation, along with detrimental changes to the gut microbiome, which can lead to broader systemic effects, including metabolic abnormalities.^1^ The complex relationship between diet, genetics, environment, and alterations in the gut microbiome are known etiological factors involved in the development of IBD.^2^ The rapid increase in the incidence and prevalence of this chronic condition in continents such as Asia, Africa, South America, and Eastern Europe could point to the vital role diet plays in disease development.^3^ In particular, the increased consumption of a Western diet pattern (WD) characterized by high n-6 polyunsaturated (n-6 PUFA) vegetable oils, processed meat, sweetened beverages, environmental contaminants, and food additives with a concomitant reduction in protective phytochemicals fiber, fruits, vegetables, and fish is associated with an increased risk of developing UC.^4^ Thereby; it appears that the WD promotes local and systemic inflammation driven by changes in gut microbiota and the immune system, affecting the gut integrity.^5^

Accumulating evidence from genetic studies indicates that UC results from an aberrant inflammatory response to intestinal microbes in a genetically susceptible host.^6^ Structural and functional defects in the intestinal mucosal barrier, such as reduced goblet cell numbers, impairments in mucin 2 (Muc2) secretion and a thinner than normal mucus layer have been found in inflamed tissues of patients with UC.^7^ As a result of these defects, microbes are in continuous contact with the intestinal epithelium triggering immune activation.^8^ Similarly, mice that lack Muc2 (Muc2^−/−^ mice), which subsequently impairs intestinal barrier function, develop spontaneous colitis which is dependent on the presence of gut microbes and environmental conditions (i.e., facility).^9,10^ Muc2^−/−^ mice display dynamic changes in the gut microbiota including enrichment in potential opportunistic pathogens *Akkermansia muciniphila*, and decreases in *Lactobacillus* spp. and Lachnospiraceae.^11^ The mucosal defense factor resistin-like molecule-β (RELM-β) is upregulated during gut inflammation and RELM-β deficiency attenuates colitis development and symptoms in Muc2^−/−^ mice. The upregulation of RELM-β in the Muc2^−/−^ model is associated with severe rectal prolapse, which is considered a severe form of colitis in this model.^12^

Much of the research on diet and IBD has focused on the negative impact of high fat intake and its association with IBD.^13,14^ However, one diet higher in the recommended fat intake than national guidelines, the Mediterranean diet (MD) (40% by energy derived from fat), has been associated with beneficial effects in immune and metabolic diseases, including IBD.^15^ Additionally, we and others have shown that the type of fat, independent of caloric content, influence intestinal inflammation, metabolism, and host-microbe function.^16,17,18^ For example, murine models have demonstrated that n-6 PUFA and saturated fatty acids (SFA) result in inflammation-induced colonic damage while monounsaturated fatty acids (MUFA) are protective. Additionally, the benefits of n-3 PUFA, commonly present in fish oils, may depend on SFA in the diet.^17^ SFA derived from dairy fat are unique in their compensatory inflammatory responses involved in tissue restitution.^16,17^ Human observational studies show that after energy-adjustment for total fat intake, myristic acid (a SFA) and long-term intake of trans-fatty acids and n-6 PUFA are associated with an increasing incidence and risk of a flare-up in UC patients.^13,14^ Understanding the mechanisms of how various fatty acids impact IBD is important in the development of evidence-based guidelines to reduce specific food-induced inflammation, promote remission and dietary tolerance.

Therefore, the primary aim of this study was to compare a MD fat blend (high MUFA, 2:1 n-6:n-3 PUFA and moderate SFA) to diets composed of corn oil (CO, n-6 PUFA rich), olive oil (OO high MUFA) or milk fat (MF, high SFA) on intestinal inflammation, glucose metabolism, and the gut microbiome in the Muc2^−/−^ model of spontaneous colitis. The fatty acid profile of the MD was designed to reflect the fatty acid profile of the MD consumed by humans in Mediterranean regions.^19^ We show that Muc2^−/−^ mice fed the MD were protected from the development of severe colitis and impairments in glucose tolerance through anti-inflammatory host defense mechanisms. Uniquely, the MD was associated with health-promoting microbes such as *Lactobacillus animalis* and Muribaculaceae.

## Results

### MD protects against severe clinical disease activity and histological damage

**Figure 1.**
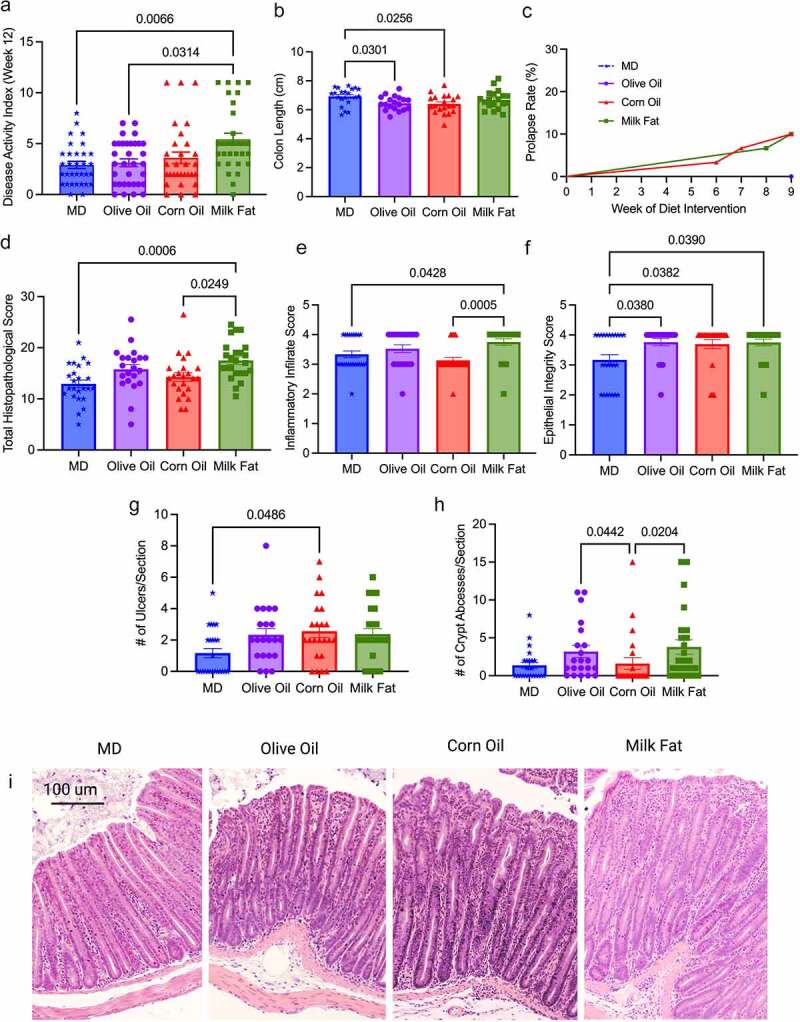
**MD protects against severe clinical disease activity and histological damage**. Colitis severity was measured by (a) disease activity index at endpoint in four diet groups: MD, olive oil, corn oil and milk fat diets, (b) colon length at endpoint, (c) rectal prolapse rate of the mice, (d) total histopathological scores, (e) inflammatory infiltrate score (f) epithelial integrity scores (g) ulcers per tissue section and (h) crypt abscesses per tissue section (i) representative H & E stained cross-sections of the distal colon (images taken at 10x magnification). Colons analyzed in (D) were scored for inflammation, ulceration, edema, epithelial integrity, and hyperplasia. *n* = 30–33 mice/group. Statistical significance was determined by Kruskal-Wallis with Dunn’s post-hoc analysis, means ± SEM. MD: Mediterranean diet; SEM: standard error of the mean. *p* *<* 0.05. (See also Figure S1).

A MD fat blend was compared to isocaloric corn oil, olive oil or milk fat diets in Muc2^−/−^ mice (Table 1). No differences in food intake or weight changes were seen between any diet groups (Figure S1A and S1B), indicating that variations in colitis were not driven by increased caloric intake or weight gain. Mice fed the MD and OO diets had significantly lower disease activity index (DAI) scores than the MF diet, with the MD showing the lowest DAI compared to the other diet groups (Figure 1a, Figure S1C). Ten percent of the CO and MF mice developed rectal prolapse, the most severe form of colitis assessed by our scoring system. No rectal prolapses were seen in the MD or OO diets (Figure 1c). The CO diet had an earlier onset of rectal prolapse (week 6, week 7 and week 9) than the MF diet (week 8 and 2 mice at week 9), suggesting the mice fed CO developed a more aggressive form of colitis earlier. Shortening of the colon, a hallmark sign of increased inflammation and disease severity in colitis,^20^ was not seen in the MD or MF fed diet, whereas the CO and OO fed diets had significantly shorter colons than mice fed the MD (Figure 1b). In agreement with the clinical observations, histopathological differences were observed between the diet groups the MD group showed the lowest total histopathological scores with the least epithelial damage, in addition to fewer ulcerations and abscesses (Figure 1i). Data from both males and females were combined, as statistically significant sex differences were not detected (Figure S1D). Overall, the Muc2^−/−^ mice fed the CO and MF diet exhibited multiple features of more aggressive colitis, whereas the MD presented with a milder form of the disease.

**Table 1.** Nutrient composition of experimental diets. Four diets were examined in this study – Mediterranean Diet, Olive Oil, Corn Oil and Anhydrous Milk Fat. All diets were isocaloric iso-nitrogenous with similar protein and carbohydrate. Lipid content was altered to reflect the various fatty acid profiles. Four diets were examined in this study – Mediterranean Diet, Olive Oil, Corn Oil and Anhydrous Milk Fat. All diets were isocaloric iso-nitrogenous with similar protein and carbohydrate. Lipid content was altered to reflect the various fatty acid profiles

	Mediterranean Diet (TD. 170674)	Olive Oil (TD. 130128)	Corn Oil (TD. 120022)	Anhydrous Milk Fat (TD. 120021)
*MACRONUTRIENTS*				
Energy Density (Kcal/g)	4.5	4.5	4.5	4.5
Protein (%) *casein*	19	19	19	19
Carbohydrates (%) *sucrose, corn starch* *maltodextrin*	40.1	40.1	40.1	40.1
Fat (%)	40.8 olive, corn, anhydrous milk fat fish oil	40.8 olive, soybean	40.8 corn, soybean	40.8 anhydrous milk fat, soybean
*FATTY ACIDS *				
Total SFA (g/kg)	38.3	34.0	28.9	125.2
Total MUFA (g/kg)	136.6	139.7	54.6	61.8
Total PUFA (g/kg)	24.7	26.3	116.3	12.8
Total MCFA (g/kg)	0.7	0	0	16.2
n-6 PUFA:n3 PUFA ratio (n:1)	2	2	46	5
4:0 Butyric (g/kg)	0.3	0	0	7.2
6:0 Caproic (g/kg)	0.2	0	0	4.4
8:0 Caprylic (g/kg)	0.1	0	0	2.1
10:0 Capric (g/kg)	0.2	0	0	3.8
12:0 Lauric (g/kg)	0.2	0	0	5.9
14:0 Myristic (g/kg)	1.3	0	0	22.2
16:0 Palmitic (g/kg)	28.4	27.0	24.3	50.9
16:1 Palmitoleic (g/kg)	2.8	2.3	0	3.6
18:0 Stearic (g/kg)	5.8	5.2	4.6	24.2
18:1 Oleic (g/kg)	133.7	137.4	54.6	55.9
18:2 Linoleic (g/kg)	21.9	24.3	113.6	10.8
18:3 Linolenic (g/kg)	1.3	1.9	2.7	1.8
20:5 EPA (g/kg)	0.5	0	0	0
22:6 DHA (g/kg)	0.4	0	0	0
*VITAMINS –* Vitamin Mix, Teklad (40060)				
Vitamin A (IU/g)	24035	23789	23789	29641
Vitamin D3 (IU/g)	2643	2643	2643	2643
Vitamin E (IU/kg)	145	145	145	145
Vitamin K3 (menadione) (mg/kg)	59.5	59.5	59.5	59.5
Vitamin B1 (thiamin) (mg/kg)	21.4	21.4	21.4	21.4
Vitamin B2 (riboflavin) (mg/kg)	26.4	26.4	26.4	26.4
Niacin (nicotinic acid) (mg/kg)	118.9	118.9	118.9	118.9
Vitamin B6 (pyroxidine) (mg/kg)	21.8	21.8	21.8	21.8
Pantothenic Acid (mg/kg)	72.6	72.6	72.6	72.6
Vitamin B12 (cyanocobalamin) (mg/kg)	0.04	0.04	0.04	0.04
Biotin (mg/kg)	0.53	0.53	0.53	0.53
Folate (mg/kg)	2.4	2.4	2.4	2.4
Choline (mg/kg)	1686.9	1686.9	1686.9	1686.9
Vitamin C (mg/kg)	1189.4	1189.4	1189.4	1189.4
*MINERALS –* Mineral Mix, AIN-76 (170915)				
Calcium (g/kg)	7.7	7.7	7.7	7.7
Phosphorus (g/kg)	6.5	6.5	6.5	6.5
Sodium (g/kg)	1.2	1.2	1.2	1.2
Potassium (g/kg)	4.3	4.3	4.3	4.3
Chloride (g/kg)	1.9	1.9	1.9	1.9
Magnesium (g/kg)	0.614	0.614	0.614	0.614
Zinc (mg/kg)	48.6	48.6	48.6	48.6
Manganese (mg/kg)	70.3	70.3	70.3	70.3
Copper (mg/kg)	7.2	7.2	7.2	7.2
Iodine (mg/kg)	0.25	0.25	0.25	0.25
Iron (mg/kg)	44.1	44.1	44.1	44.1
Selenium (mg/kg)	0.13	0.13	0.13	0.13
Calcium carbonate (g/kg)	3.6	3.6	3.6	3.6
DL methionine (g/kg)	3.6	3.6	3.6	3.6
Food color (g/kg)	0.1 blue	0.1 orange	0.1 red	0.1 green

^a^pelleted and irradiated

Abbreviations: SFA: saturated fatty acids; MUFA: monounsaturated fatty acids; PUFA: polyunsaturated fatty acids; MCFA: medium chain fatty acids; EPA: eicosapentaenoic acid; DHA: docosahexaenoic acid

**Figure 2.**
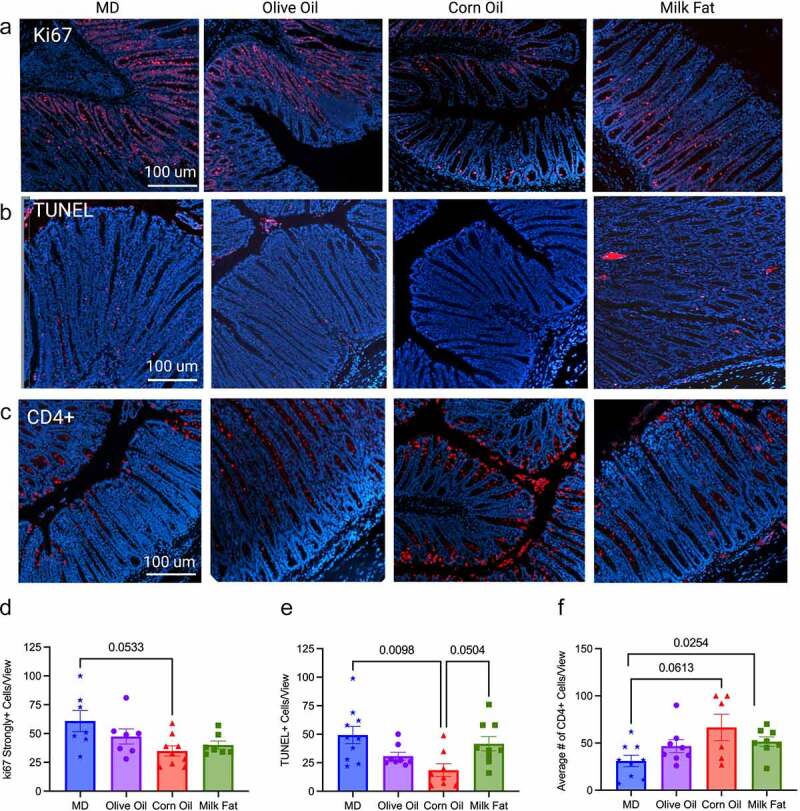
**MD maintains tissue homeostasis through control of Ki67 and apoptosis**. (a) Ki67 strongly positive cells seen as red fluorescence with DAPI used as a counterstain in the MD, olive oil, corn oil and milk fat diets, (d) quantification of the strongly positive Ki67 cells per view are shown. (b) Distal colon sections were stained for apoptotic cells using the TUNEL assay, strongly positive cells seen as red fluorescence with DAPI used as a counterstain and (e) the quantification number of apoptotic cells per view. (c) immunostaining for CD4 + T-cells seen as red with DAPI used as counterstain and (f) the average number of CD4 + T-cells per view. *n* = 8–10 mice/group. (scale bars: 100 µm, magnification, 100x). Statistical significance was determined by Kruskal-Wallis with Dunn’s post-hoc analysis, means ± SEM. MD: Mediterranean diet; TUNEL: terminal deoxynucleotidyl transferase dUTP nick end labeling; SEM: standard error of the mean. *p* *<* 0.05. (See also Figure S2).

Given the histological differences seen between the diet groups, we next examined homeostatic responses, including the endogenous nuclear protein Ki67, as a marker of cell proliferation through immunofluorescent staining of the colonic intestinal epithelial cells. The MD had the highest Ki67 positive cells compared to CO (Figure 2a and 2d). Dysfunctions in apoptosis (programmed cell death) are implicated in IBD pathogenesis.^21^ In the colon, differentiated cells are in a constant state of renewal balanced between proliferation and cell death to maintain homeostasis of the intestinal barrier.^22^ We examined apoptosis using the terminal deoxynucleotidyl transferase dUTP nick end labeling (TUNEL) assay for apoptotic DNA fragmentation in the cypts of the distal colon (Figure 2b and 2e). The MD and MF diets show more cells positive for apoptotic DNA fragmentation than the CO diet. These results suggest the CO diet has a dysregulated balance between proliferation and apoptosis.

Neutrophil infiltration was assessed through immunofluorescent staining of myeloperoxidase positive (MPO+) cells, as an over-exuberant neutrophil response is known to cause epithelial damage in IBD.^23^ The MF and OO fed diets had the highest number of MPO+ cells, with the MD showing significantly less infiltration than the MF diet (Figure S2). Unexpectedly, the CO-fed diet had weak staining for MPO+ cell infiltration compared to the other diet groups despite showing more ulcerations and more severe colitis, which suggests other intestinal responses besides neutrophils are driving colonic damage, although we did not rule out netosis. Immunostaining for T lymphocytes confirmed a significant influx of CD4 + T lymphocytes into the intestinal mucosa of the CO and MF-fed diets in comparison to the MD (Figure 2c and 2f). CD4 + T cells are enriched in lesional tissue and are key initiators of disease progression in colitis.^24^ The increased damage seen in the CO diet may result from suppression of important homeostatic immune responses (proliferation and apoptosis) and increased infiltration of CD4 + T lymphocytes. Overall, the fat blend in the MD protects against severe and damaging colitis due to functional homeostatic balance.

### MD decreases colonic cytokines that drive colitis

**Figure 3.**
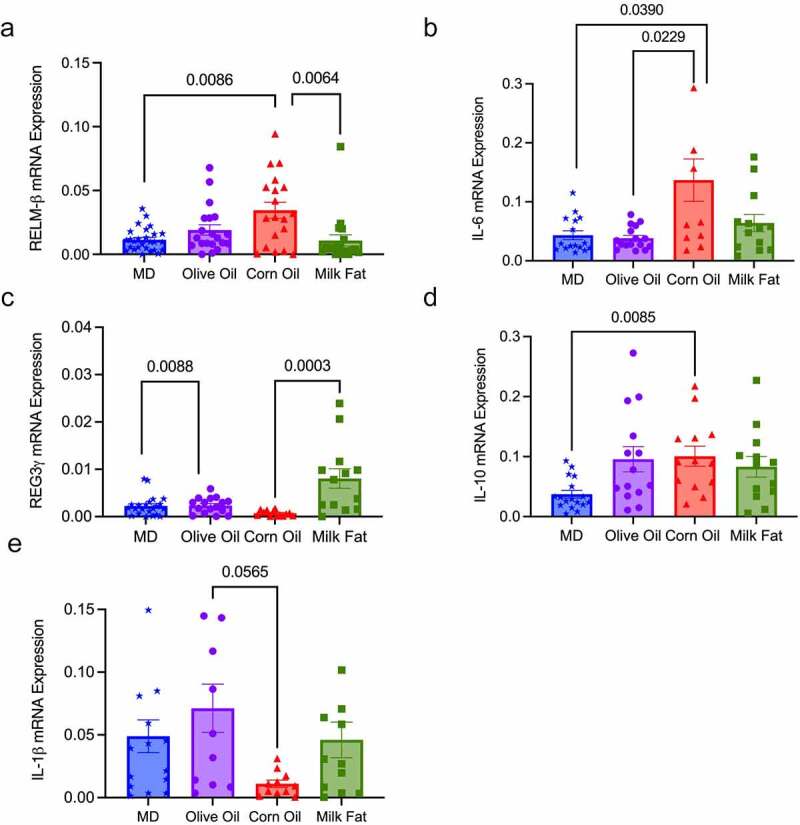
**MD decreases colonic mRNA expression of pro-inflammatory cytokines RELM-ß and IL-6**. Relative colonic mRNA expression (a) RELM-β, (b) IL-6 (c) REG3-γ, (d) IL-10 and (e) IL-1β. *n* = 20–26 mice/group. Statistical significance was determined by Kruskal-Wallis with Dunn’s post-hoc analysis, means ± SEM. MD: Mediterranean diet; standard error of the mean. *p* *<* 0.05. (See also Figure S3).

To determine how the MD fat blend protects against the development of severe colitis, we examined the cytokine mRNA gene expression in the distal colon, specifically RELM-ß and IL-6, as they are known drivers of colitis in the Muc2^−/−^ model.^12^ In accordance with the clinical markers of disease and the histological analysis, we saw a significant decrease in the expression of mRNA inflammatory cytokines RELM-ß and IL-6 in the MD compared to the CO diet (Figure 3a and 3b). Interestingly, despite the increased DAI and histological damage seen in the MF diet, the MF diet uniquely showed reduced mRNA expression of RELM-ß, yet an increased expression of the mRNA antimicrobial peptide REG3-γ (Figure 3a and 3c). No differences were seen between TNF-α, FOXP3, TGF-β1, Ebi3 or IL-22 colonic mRNA expression amongst the diet groups (Table S3). Taken together, the MD reduced the expression of the colonic mRNA pro-inflammatory cytokines that drive colitis.

### MD stimulates Th 22 cells important in maintaining epithelial homeostasis

**Figure 4.**
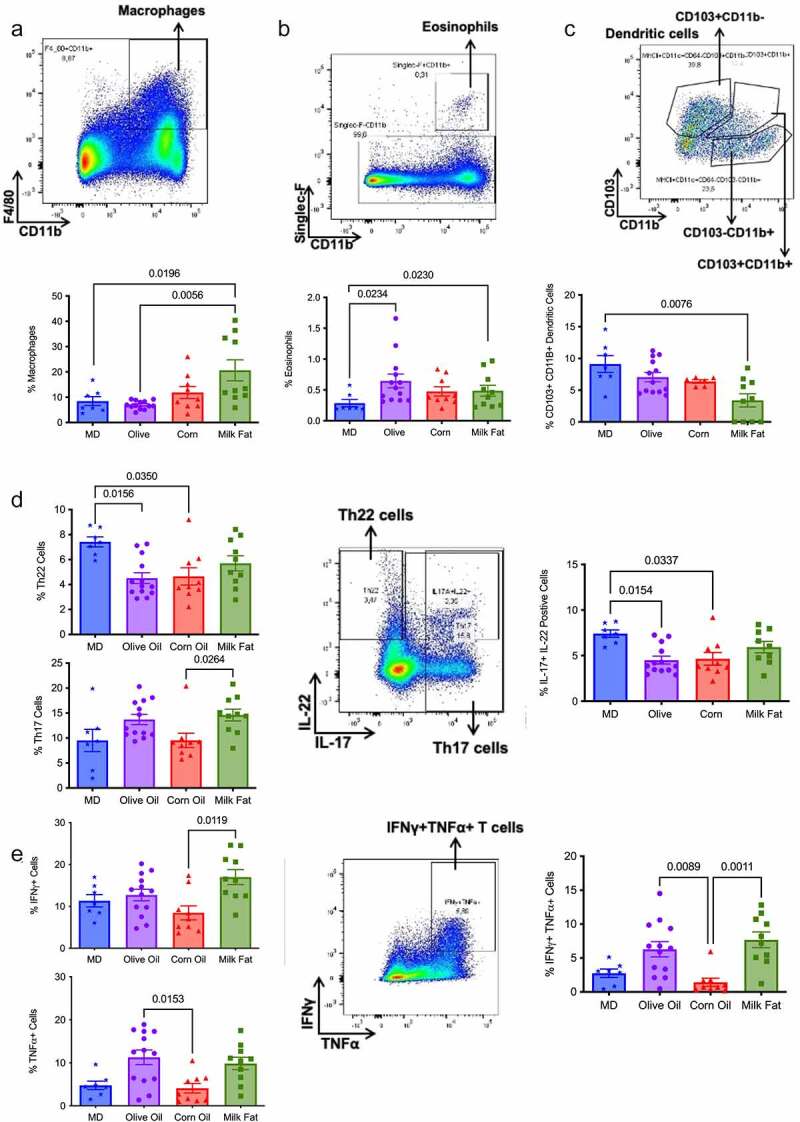
**MD stimulates tolerogenic CD103^+^ dendritic and Th22 cells vital in maintaining epithelial homeostasis**. Flow cytometry analysis of the populations of intestinal lamina propria cells and their cytokines produced in the distal colon. The proportion of (a) macrophages (F4/80+ CD11b+), (b) eosinophils (Siglec-F CD11B+), (c) dendritic cells (CD103+ CD11b+), (d) Th22, Th17 and Il-17+ IL22+ cells, (e) IFNγ+ cells, TNFα cells and IFNγ+TNFα+ cells. *n* = 7–10 mice/group. Statistical significance was determined by Kruskal-Wallis with Dunn’s post-hoc analysis, means ± SEM. MD: Mediterranean diet SEM: standard error of the mean. *p* *<* 0.05. (See also Figure S4).

Immunophenotyping was completed to determine the relative abundance of different immune cells found in the lamina propria of the colon. There were no significant differences between the diets in the proportion of inflammatory monocytes and neutrophils (Table S4); however, the MF diet did show the largest absolute number of these cells, which correlates with the increased ulcers and abscesses seen in the histology. There were significant increases in macrophages in the MF diet compared to the MD and OO diets (Figure 4a), with significantly lower numbers of eosinophils in the MD compared to OO and MF diets. (Figure 4b). Eosinophils have been found in the inflamed tissue of colitis patients, leading to increase diarrhea, inflammation, and tissue destruction promoted by the action of IL-5.^25,26^ We saw reduced eosinophils and serum IL-5 (Table 2) in the MD in comparison to the mice fed CO, further demonstrating the protective immunomodulatory effects of a fat blend (MD), favoring a milder colitis phenotype.

**Table 2.** MD stimulates key cytokines G-CSF and MCP-1 necessary for a balanced immune response.^a^  Serum samples were collected at the end of the study. The level of cytokine production in the serum was measured using addressable laser bead immunoassay. (See also Figure S5)

Serum Cytokines Mean ± SEM, by Diet					
	MD (n = 7)	Olive Oil (n = 8)	Corn Oil (n = 7)	Milk Fat (n = 7)	*P* Value^a^
G-CSF	3066 ± 397^b^	3097 ± 1040	1458 ± 411	2668 ± 673	0.0221 (CO vs MD)
MCP-1	78.4 ± 17.3	59.8 ± 8.30	29.6 ± 6.90	40.8 ± 8.42	0.0238 (MD vs CO) 0.0129 (CO vs OO)
IL-5	9.27 ± 2.03^b^	20.2 ± 5.81	19.1 ± 2.94	22.6 ± 7.94	0.0321 (MD vs CO)
IL-10	20.1 ± 3.81	9.36 ± 1.21^b^	21.7 ± 3.82	15.6 ± 4.14	0.0192 (MD vs OO) 0.0076 (CO vs OO)
IL-17	22.4 ± 5.95	17.6 ± 2.83	11.7 ± 2.40	28.7 ± 7.22	0.0262 (CO vs MF)
INF-γ	5.98 ± 2.61	12.6 ± 2.28^b^	4.40 ± 1.78	12.6 ± 2.28	0.0105 (CO vs OO)

*^a^*By the Kruskal-Wallis test with Dunn’s multiple comparisons test. A value of *p* < 0.05 was considered statistically significant. ^b^One sample detected as out of range of the standard curve.

Dendritic cells (DC), in particular, tolerogenic CD103^+^ DCs, are crucial for intestinal homeostasis as they prevent aberrant immune responses.^27^ MD resulted in a significant increase in the tolerogenic CD103+ CD11b+ DCs versus the MF group (Figure 4c). We next examined the various immune cell subtypes to elucidate further how the type of fat impacts the adaptive inflammatory responses. Th17 and Th22 cells, along with serum IL-17 and MCP-1 (Table 2), play a protective role in IBD promoting barrier function through epithelial cell regeneration, host protection through immune cell recruitment and maintaining intestinal homeostasis.^28,29^ Within the T helper (Th) cell populations, Th22 cells and IL-17+ IL-22+ cells were significantly increased in the MD versus the OO and CO fed diets (Figure 4d). Th22 cells and IL-17+ IL-22+ cells promote barrier repair and epithelial homeostasis.^29^ Surprisingly, the CO diet had the most colonic damage and increased colonic CD4 + T cells, yet we observed a reduced proportion of Th17 and IFNγ+TNFα+ producing cells in the lamina propria (Figure 4e) with concomitant reductions in serum IL-17 and IFN-γ levels (Table 2). Further examination of the systemic cytokines indicates that the CO-fed mice had reduced serum production of granulocyte colony-stimulating factor (G-CSF) and monocyte chemoattractant protein-1 (MCP-1) when compared to the MD fed mice (Table 2). G-CSF is a crucial regulator of neutrophil differentiation and enhances the bactericidal function of neutrophils, whereas MCP-1 is a necessary component of the inflammatory response required for tissue protection, remodeling, and healthy expansion.^30^ Mice lacking G-CSF are more susceptible to DSS-induced colitis.^31^ This would support the weak staining for MPO+ cell infiltration for neutrophils with immunofluorescent staining in the CO diet compared to the other diet groups (Figure S2). Colonic mRNA expression of IL-10 and serum IL-10 was significantly increased in the mice fed the CO diet (Figure 3d and Table 2). IL-10 is considered an essential anti-inflammatory cytokine crucial in the maintenance of immune homeostasis in IBD; however, there is emerging evidence that IL-10 may play a previously underappreciated dual role, with its function highly dependent on the timing of IL-10 production.^32^ Our data show that IL-10 mRNA expression was significantly upregulated in the CO diet compared to MD. In this scenario, as we discovered severe intestinal damage and more severe colitis in the CO diet, this could indicate a dysfunctional compensatory effect of IL-10 in this diet group, given the associated damage in the colon. No differences in additional chemokines or cytokines were noted (Table S5). Overall, the MD fat blend was more protective against dysfunctional immune responses than any fat alone. Although the MD did experience inflammation, the inflammation was associated with less damage suggesting these responses were protective in the Muc2^−/−^ mice lacking the mucus layer.

### Milk fat improves glucose homeostasis, intestinal permeability, and barrier function

**Figure 5.**
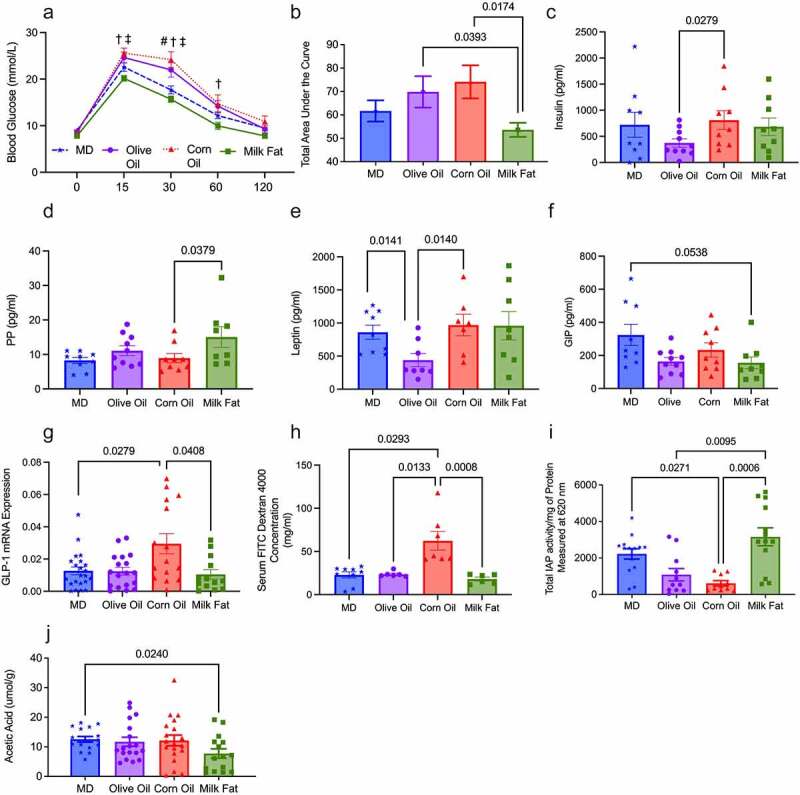
**Saturated fat, derived from MF, improves glucose homeostasis, intestinal permeability, and barrier function**. (a) Intraperitoneal glucose tolerance test (IPGTT) for all diet groups (*n* = 12–19 mice/group) was performed at the end of the study, (b) along with the corresponding area under the curve (AUC). Serum concentrations of hormones (c) insulin, (d) PP (e) leptin and (f) GIP (*n* = 9–10/group). (g) mRNA expression of GLP-1. (h) Fluorescein isothiocyanate–dextran 4kDa (FITC) measured intestinal permeability (*n* = 6/group). (i) The key mucosal defense factor, intestinal alkaline phosphatase (IAP), was measured in the colon (*n* = 11–14 group). Short-chain fatty acid (j) acetic acid was measured in the cecal content (*n* = 15–19 mice/group). MD: Mediterranean diet; PP: pancreatic polypeptide; GLP-1: glucagon-like peptide-1; GIP: gastric inhibitory peptide; SFA: saturated fatty acids; MUFA: monounsaturated fatty acids; n-6 PUFA; n-6 polyunsaturated fatty acids; n-3 PUFA: n-6 polyunsaturated fatty acids; SEM: standard error of the mean. ^§^*p < *.05 than milk fat; ^‡^*p* *<* 0.05 than corn oil; #*p* *<*0 .05 than MD; and ^†^*p* *<* 0.05 than olive oil. (See also Figure S6).

As Muc2^−/−^ mice develop metabolic dysfunction,^18^ we conducted an intraperitoneal glucose tolerance test (IPGTT) to investigate how the MD fat blend impacts glucose tolerance compared to the individual fat diets. Baseline fasting glucose levels were not different between the various diet groups (0 minutes in IPGTT, Figure 5a); however, upon glucose challenge, the mice fed CO and OO diets had significantly higher glucose levels than the MD and MF diets at multiple time points (Figure 5a). These results were confirmed by calculating the area under the curve (Figure 5b), which indicates that dietary fats have differential effects on glucose clearance.

Serum metabolic hormone levels revealed the OO diet had significantly lower serum insulin levels than the CO diet (Figure 5c), and pancreatic polypeptide (PP) was significantly increased in the MF diet versus the CO diet (Figure 5d). Leptin was significantly lower in the OO diet compared to the MD and CO diet (Figure 5e). The MD had significantly higher serum levels of glucose inhibitory peptide (GIP) than the MF diet (Figure 5f). Colonic mRNA gene expression of glucagon-like peptide 1 (GLP-1) demonstrated a significant increase in expression in the CO diet when compared to the MD and MF diets (Figure 5g); however, no changes were observed in serum GLP-1, which is likely a compensatory signal to promote the increased synthesis of serum GLP-1. No differences were seen in the serum levels of glucagon, amylin, peptide YY and C-peptide, ghrelin, resistin or GLP-1 between the diet groups (Table S6). In summary, serum insulin levels between the MD, MF and CO diet are similar yet result in different glucose responses. Despite the consistent insulin levels between the MD, MF and CO diets, the CO diet requires more insulin to maintain euglycemia than the other groups; therefore, it is plausible that a CO diet-induced insulin resistance. To further support this, MCP-1 deficiency in the CO diet further contributed to metabolic perturbations, consistent with previously reported findings,^30^ where MCP-1 depletion has been linked to high-fat diet pathologies, including metabolic dysfunction fibrotic adipose tissue. In contrast to CO, we see impaired glucose homeostasis in the OO diet due to reduced serum insulin and leptin secretion. Glucose homeostasis is closely regulated by both insulin and leptin, with decreased leptin potentially contributing to reduced insulin sensitivity in the OO diet.^33^

We sought to determine if the alterations in immune responses and metabolism resulted from changes in barrier function since increased permeability can lead to endotoxemia and precedes colitis. Using the FITC dextran assay, we demonstrate that the CO diet had increased intestinal permeability (Figure 5h). We next examined intestinal alkaline phosphatase (IAP) activity since IAP activity plays a significant role in maintaining intestinal homeostasis and protection with reduced activity associated with worsened mucosal inflammation and metabolic syndrome.^34^ The MD and MF diets show increased IAP activity than the CO and OO diets (Figure 5i), suggesting that saturated fat plays a key role in IAP function.

Short-chain fatty acids (SCFA), including acetic, propionic, and butyric acid, have important immunomodulatory properties and promote gut homeostasis,^35^ we examined the cecal production of SCFA. No significant differences were seen in total SCFA, butyrate, propionate, valeric, iso-valeric or iso-butyric acid (Figure S6). However, the MF diet did show reduced production of acetic acid in comparison to the MD (Figure 5j). Although further work is needed to elucidate the influence of acetic acid on intestinal inflammation, preliminary evidence suggests that reduced levels of acetic acid could enhance susceptibility to colitis.^36^

In summary, the results presented above indicate that type of fat influences glucose homeostasis, serum hormones, IP, SCFA and IAP activity differentially. Diets that contain saturated fat, such as the MD and MF diets, demonstrate improvements in glucose metabolism, which IPGTT supports, serum hormones, increased expression of IAP and improved intestinal permeability. However, diets rich in CO and OO contribute to impairments in metabolism as seen by alterations in glucose tolerance, reduced expression of IAP and the CO diet also showing impaired intestinal permeablity. Overall, the MD fat blend containing SFA is an important part of the beneficial effects of the diet metabolism in a colitis susceptible host.

### The MD is associated with unique changes in the gut microbiota composition

The emerging evidence on the interconnectedness between the gut microbiome and host metabolism,^1^ as well as the hypothesized role of the microbiome in the etiology of IBD, led us to investigate how the MD fat blend influenced the composition of the gut bacteriome in Muc2^−/−^ mice compared to the individual fat diets. No differences in alpha diversity (Richness, Shannon-index, Pielou’s evenness and Faith’s phylogenetic diversity) or beta-diversity (Bray-Curtis) were detected in the baseline stool (Figure S7); therefore, the impact of dietary fat on the microbial composition on the gut bacteriome was examined using colon samples. Mucosal microbiota play an important role in mucosal immunity through continuous contact with intestinal-related lymphoid tissue, therefore we decided that colonic tissue would most accurately reflect the microbiota composition.^37^

**Figure 6.**
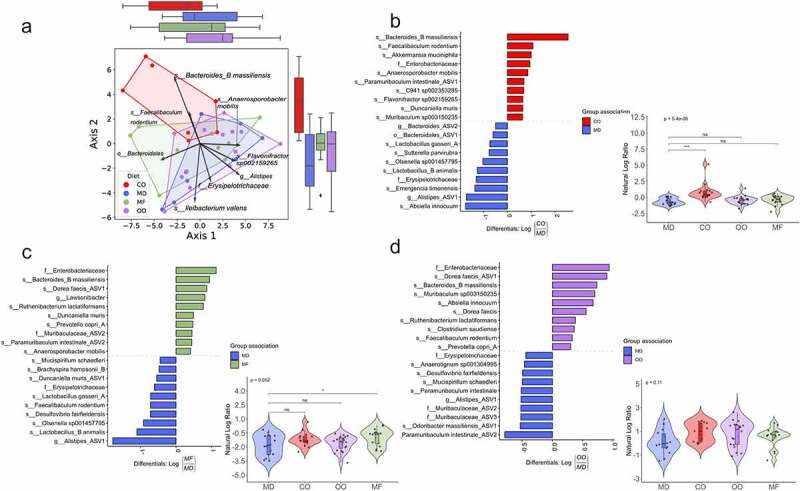
**The MD is associated with unique changes in the gut microbiota composition**. Colon samples were collected post-diet intervention and sequenced for 16S rRNA at the V4-V5 region. (a) Principal component analysis biplot of robust centered-log ratio transformed distances. Circles represent individual mice, vectors represent ASV loadings, and the diet groups are enclosed with convex hull polygons. The ridge boxplots represent the principal component values along the corresponding axis. An overall significant difference between groups was assessed using a PERMANOVA test (*p* = 0.003, Pseudo F = 3.6, permutations = 999), and the assumptions of heterogeneous dispersion were checked using a PERMDISP test (*p* = 0.769, Pseudo-F =0 .77, permutations = 999). Pairwise post-hoc testing shows a significant difference between the CO and MD groups and CO to OO groups. (B to D) Ranked plots of the inverse additive log-ratio transform (inverse ALR) differentials from Songbird’s multinomial regression analysis, which estimates the probability of an ASV being observed for a specific diet group. The top and bottom 10 ranked ASVs are displayed as their highest classified taxonomic level based on the GTDB reference database. A positive value indicates a higher association with the numerator group. A negative value suggests a higher association with the denominator group. The MD is used as the reference group compared to CO, MF and OO diets (n = 7–14 mice per group). (See also Figure S7). ASV: amplicon sequence variant; GTDB: gene taxonomy database; PERMANOVA: permutational multivariate analysis of variance; PERMDISP: permutational analysis of multivariate dispersions.

**Figure 7.**
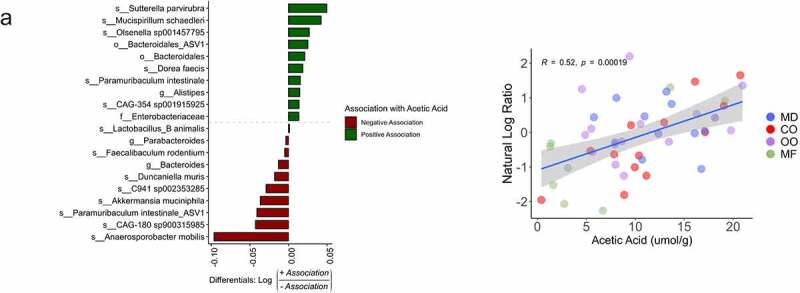
Microbial taxa associated with acetic acid. Top 10 ranked microbial taxa associated with acetic acid in the MD and MF diet groups (n = 8–12 mice per group). Associations determined using a Songbird model (ASVs, diet + acetic acid) validated with diet as the explanatory variable. Pearson correlation r = 0.52, p =0 .00019). ASV: amplicon sequence variant.

β-diversity using the compositionally-aware robust Atchison distance detected differences between diet groups using a PERMANOVA test (p = .003, pseudo-F = 3.6, permutations = 999) (Figure 6a). A post-hoc pairwise test of the PERMANOVA results revealed that the CO group was statistically significant from the MD and OO and appreciably different than MF, though this was not statistically significant (Table S8C). The top ASVs associated with the MD were *Absiella innocuum, Alistipes *sp*., Emergencia timonensis* and *Lactobacillus animalis*, whereas *Blautia massiliensis, Faecalibaculum rodentium, Akkermansia muciniphila, Anaerosporobacter mobilis* were the top-ranked taxa associated with the CO diet (Figure 6b). When the MD was compared to the MF diet, the top ranked taxa associated with the MD were *Alistipes* spp., *Lactobacillus animalis, Olsenella* 001457795, *Desulfovibrio fairfieldensis* and *Faecalibaculum rodentium* while in the MF diet *Blautia massiliensis, Dorea faecis, Lawsonibacter* spp. and *Ruthenibacterium lactatiformans* were most abundant (Figure 6c). The top-ranked taxa in the MD compared to OO diet were *Paramuribaculum intestinale, Odoribacter massiliensis*, Muribaculaceae, and *Alistipes* spp. whereas *Dorea faecis, Bacteroides massiliensis, Muribaculum* sp003150235, *Absiella innocuum* were the top-ranked taxa associated with the OO diet (Figure 6d). Overall, there was a consistent association with ASVs from the family of Enterobacteriaceae and *Bacteroides massiliensis* in all the fat diets compared to the MD. The effect of the CO diet on different taxa was most pronounced out of all the diet groups, with OO being most similar. Notably, we identified key taxa associated with the changes in acetic acid. Higher acetic acid levels were positively correlated with the ASVs, *Sutterella parviruba, Mucispirillum schaedleri, Olsenella sp001457795* and Bacteriodales and negatively correlated with *Anaeosporobacter mobilis, CAG-180* sp900315985, *Paramuribaculum intestinale* and *A. muciniphila* (Figure 7). The ratio of these positively associated ASVs to negatively associated ones were higher in the MD group compared to MF. No further correlations were found between SCFAs and ASVs for the other diet groups. Together, these results confirm that various types of fat alter the composition of the gut microbiota.

## Discussion

The role of diet in IBD is underappreciated, yet evidence-based diet recommendations are needed to determine which dietary patterns IBD patients can tolerate in their everyday lives. Despite the increasing evidence of the influence of nutritional components and their impact on disease activity, inflammation and the microbiome, significant gaps still exist. This mechanistic study provides compelling evidence that type of fat, not total calories derived from fat, impacts the gut microbiota composition, inflammation, and disease activity in a murine model (Muc2^−/−^) that develops spontaneous colitis with the MD fat blend protecting against severe colitis.

We demonstrate that the MD, created by blending high levels of MUFA (from OO), SFA (from MF) combined with some n-3 PUFA (from fish oil) with low n-6 PUFA (from CO), creates a protective fat combination against colitis. Mice fed the MD fat blend were protected from developing severe colitis demonstrated by reduced disease activity histological damage in the distal colon, including fewer ulcers and abscesses compared to the other diet groups. In contrast, mice fed a diet composed of CO or MF develop severe colitis with increased mucosal damage. Although the MD is rich in OO, OO alone resulted in the Muc2^−/−^ mice developing colonic damage, albeit less severe than that seen in the CO or MF diets which both resulted in severe colitis. This data exemplifies that the MD fat blend, not a single type of fat, is important in its ability to protect against colitis completely.

Each type of fat demonstrates unique immunological responses from a local and systemic level. Unique to the MD, we see a significant increase in the proportion of Th22 cells and tolerogenic CD103+ CD11b+ dendritic cells and immune regulating signals like serum G-CSF, which are critical in mediating tolerance to antigens, limiting reactivity to the gut microbiota and are required for restitution.^24,27,31^ The MF diet showed compensatory protective responses, such as the increased activity of the key mucosal defense factor IAP and antimicrobial lectin RegIII-γ that promotes restitution important in preventing the cycle of chronic inflammation.^12,38^ These data suggest that the inflammatory response, as seen in the MD, is counterbalanced by an immunosuppressive response, limiting colonic damage. When MF is provided within a blend of fats, as seen in the MD, the protective inflammatory responses of the MF diet are conferred, limiting mucosal damage and ultimately colitis severity.

We observe an increased mRNA expression of RELM-β with an increased rectal prolapse rate in the CO diet. In contrast, the MF diet had lower RELM-β expression compared to the CO yet displayed a colitic phenotype; however, these diets resulted in very different metabolic phenotypic profiles. Thus, the hypothesis that RELM-β drives susceptibility to colonic inflammation in the Muc2^−/−^ model may depend on additional factors, including diet.^39^ Mice fed a high-fat diet (lard as a saturated fat source) exhibit higher levels of RELM-β protein in the stool and have altered glucose intolerance and hyperlipidemia due to impaired insulin signaling.^39,40^ Notably, our results from the MF diet, a different type of saturated fatty acid, contradict these findings, which could suggest that different types of saturated fatty acids may influence RELM-β expression. We also observed the upregulation of RegIII-γ in the MF diet, which plays a crucial role in barrier function and may provide insight into why we see an improved metabolic profile in the MF diet. Further research is needed to determine how various types of fat may influence RELM-β and RegIII-γ expression.

Dietary fat composition has been implicated in the development of insulin resistance, including type 2 diabetes, with each type of fat having vastly different effects on insulin resistance and metabolic control.^41^ Metabolic abnormalities are increasingly being identified in both human and animal models of colitis.^1^ We demonstrate in Muc2^−/−^ mice that OO and CO diets promote impairments in glucose tolerance, barrier function and alterations to hormone levels leading to metabolic alterations. The MD and MF diet, composed of SFA, demonstrate improved glucose tolerance and barrier function. In line with the unique immunological effects of each type of fat, these results show that MF provided as a component of a fat blend (MD) plays a key role in maintaining glucose homeostasis and gut barrier function. The potential mechanisms could be through increased activity of the key mucosal defense factor IAP,^34^ reduced expression of RELM-β and increases in key hormones like PP known to enhance insulin sensitivity.^42^

Alterations in the microbiota are linked to IBD, metabolic diseases, as well as other inflammatory conditions.^1^ Multiple research groups have established the effect of a high-fat diet (i.e., the total calories derived from fat) on the microbiome,^43^ however knowledge on the influence of specific types of fat on gut microbial ecology is lacking. Our analyses demonstrate that the type of dietary fat influences the microbiota composition and is associated with microbes that are associated with health. The top-ranked taxa associated with the MD consisted of ASVs classified as *Absiella innocuum, Lactobacillus animalis, Emergencia timonensis* and *Alistipes* spp. Among these *L. animalis* is a well-known beneficial microbe that contributes to immune modulation, epithelial adherence, enhancement of gut barrier function and anti-tumorigenic potential,^44^ while *Alistipes* spp. has been associated with less severe colitis.^45^ Uniquely, the MD and the OO diets had a higher ratio of ASVs from the family of the health-promoting microbe Muribaculaceae which has been negatively associated with chronic diseases of the industrialized world^46^ In contrast, the top-ranked ASVs in the CO, MF and OO diets were *B. massiliensis*, with the CO diet also containing *A. muciniphila* both of which have been associated with colitis and colorectal cancer in humans and animal models.^47,48^*A. muciniphila* has pathobiont characteristics in the context of colitis.^49^ *A. muciniphila* are known mucin-degraders, combined with a disrupted mucus layer as seen in the Muc2^−/−^ mouse model, it is likely pathobiont that can penetrate the mucosal layer resulting in mucosal damage.^50^ The higher ratio of Enterobacteriaceae in the CO, MF and OO diets and *Dorea faecis* observed in the MF and OO diets may have also contributed to colitis, as others have seen an enrichment in these microbes in spontaneous colitis as they are known to promote chronic intestinal inflammation.^51,52^ The finding that increased ratios of ASVs of both mucin-degrading and pro-neoplastic bacteria are clinically important in IBD, as the risk of colorectal cancer increases when exposed to prolonged chronic intestinal inflammation.^53^ While not shown here, we postulate that microbial taxa associated with the changes in acetic acid production (e.g., *Mucispirillum schaedleri, A. muciniphila)* could contribute to the reduced intestinal inflammation seen in the MD.^51,54^ While further studies are needed, possible mechanisms for this could be through acetate’s role in improving intestinal permeability,^55^ as well as the suppression of IL-6 mRNA expression.^56^

A limitation of this study is that it was conducted in a mouse model in a specific-pathogen-free environment, limiting the microbiome’s role in causality of the phenotypes observed here. Differences in gut microbiota composition exist between humans and rodents; therefore, the precise role of type of fat and its role in colitis and the gut microbiota composition needs to be confirmed in human studies. Mechanistic investigations around dietary fats and their influence on colitis and its metabolic co-morbidities should be further investigated.

In summary, this study shows that a diet with a fat-blend, as seen in a Mediterranean diet, significantly reduces disease activity, inflammation-related biomarkers and improves metabolic parameters in the Muc2^−/−^ mouse model. In addition, we show that each type of fat differentially impacts the development of colitis and that it is not necessarily the total fat content of the diet that aggravates colitis. Although the observed effects of a MD need to be confirmed through interventional studies in individuals living with IBD, it would be prudent to have patients focus on the types of lipids and their subsequent food sources versus the restriction of total fat in the diet. The MD is a healthful diet that has the potential to maintain an appropriate immune response while mitigating the damaging effects of chronic inflammation, and future research is needed to confirm these results in humans.

## Materials And Methods

### Animal models

Mucin 2 deficient (Muc2^−/−^) mice with a C57BL/6 wild-type background were originally obtained from the Vancouver Gastrointestinal Disease Research Program (B. Vallance lab, Vancouver, BC, Canada) bred at UBC Okanagan and backcrossed with Charles River C57BL/6 mice. Mice were sex-matched and housed in sterilized, filter-topped cages, handled in a biological safety cabinet in a specific pathogen-free environment with sentinels used to test for common pathogens. All mice had free access to acidified tap water (pH of ~2.3 via the addition of HCl) and were fed irradiated food in a temperature-controlled room (22°C) with a controlled reverse lighting cycle (12-hour dark/light cycle). The animal experiment was approved by the University of British Columbia’s Animal Care Committee (Protocol No: A15-0240) in accordance with the Canadian Council on Animal Care Standards.

### Experimental set-up

Muc2^−/−^ mice were weaned at the age of 19 days, pooled into cages and randomly divided into four diet groups which only differed by fat composition (40.8% total calories derived from corn oil, olive oil, anhydrous milk fat or a Mediterranean Diet like fat blend (Teklad Envigo, Madison, WI) (Table 1). To reduce cage effects, the mice of the same sex were pooled into cages (*n* = 3–4 mice/cage). Paired breeding (1 male and 1 female) was used to mitigate husbandry effects, with the male staying with females and pups after birth. Upon weaning, pups were equally divided into cages, mixed with mice weaned from another litter, so there was a mix of mice from different breeder pairs in each cage. Our total sample size ranged from 30–33/diet group (15–19 males/diet group and 13–15 females/diet group). Environmental cage effects were mitigated by monitoring well-being and environmental conditions (e.g., light, temperature, relative humidity) daily without disturbing the cage. Fight wounds, which are indicative of aggressive behavior, were not observed. Each cage had 67.6 inches of floor space, covered in woodchips, cotton nesting, and Nestlets for cage enrichment. Once per week, body weight and clinical scoring were completed at the same time of day. To evaluate the clinical disease activity of colitis, body weight, stool consistency, rectal bleeding was recorded, and a disease activity score was calculated as previously described.^57^ At day 84, the mice were euthanized, and the tissues excised (colon, cecum, liver), flash-frozen in liquid nitrogen, immersed in 10% formalin (Fisher) or RNAlater (Qiagen). Until further analysis, flash-frozen tissues and those stored in RNAlater were stored at −80°C until further investigation.

### Histopathological scoring

Colon tissues were embedded in paraffin and stained with hematoxylin and eosin by Wax-it Histology Services Inc., (Vancouver, BC, Canada). Coded samples blinded by the scorers were evaluated and scored by at least two people. The scoring of colonic inflammation was quantified using a combination of approaches as previously described^58,59^

### Immunofluorescence

Paraffin-embedded colon tissue sections were deparaffinized in xylene and progressively rehydrated in decreasing ethanol concentrations (100, 90, 80 and 70% for 3 min each), and finally incubated in de-ionized water for 3 min. The Ag retrieval process was performed by incubating the slides a 1 mg/mL trypsin (Sigma) for 30 min followed by a 5% BSA blocking solution. The sections were then incubated for 2 hours at room temperature using: rabbit polyclonal antibody-1 for myeloperoxidase (Invitrogen) to examine neutrophils; rat monoclonal antibody for F4/80 (CedarLane) to examine macrophages and rabbit monoclonal antibody for Ki-67 (CedarLane) for cellular proliferation. Secondary antibodies used include goat anti-rabbit IgG AlexaFluor-conjugated 594-red antibody (Invitrogen) or goat anti-rabbit IgG 488-conjugated antibody (Invitrogen). Tissue sections were mounted using fluoroshield with DAPI (Sigma) and viewed on an Olympus IX81 fluorescent microscope. For inflammatory cell counts, positive cells were quantified by two blinded observers under fluorescence using Olympus cellSens Software, followed by further quantification by Image J.

### TUNEL assay

Apoptotic DNA fragmentation was examined using 30064 CF™ 594 Dye TUNEL Assay Apoptosis Detection Kit (Biotium, Cedarlane) according to the manufacturer’s protocol. Briefly, paraffin-fixed colon tissues were deparaffinized according to standard protocols. Cells were permeabilized in 20 ug/ml proteinase K in PBS at 37°C for 30 minutes, incubated for 5 minutes with 100 ul of TUNEL Equilibration Buffer, followed by TdT enzyme with TUNEL Reaction Buffer and labeled with fluorescein 594-dUTP using terminal deoxynucleotidyl transferase. The localized red fluorescence of the apoptotic cells was absorbed using Olympus cellSens Software.

### RNA extraction and quantitative real-time PCR (qPCR)

Upon excision, colon and liver tissues were placed in RNAlater (Qiagen) and stored in the −80°C freezer until extraction. According to the manufacturer’s instructions, RNA was extracted from the tissues using the Qiagen RNeasy Fibrous Tissue Mini kit (Qiagen). Total RNA was quantified using a NanoDrop 2000c Spectrophotometer (Thermo Scientific) and cDNA synthesized with iScript cDNA Synthesis Kit (Bio-Rad). Quantification of cDNA was performed on a Bio-Rad CFX Manager 2.0 machine using Sso Fast Eva Green Supermix (Bio-Rad). All primers were synthesized by Integrated DNA Technology, Canada (Table S8). Gene expression was normalized to TBP mRNA level and calculated as ∆Ct = 2(CtTBP mRNA – CTgene of interest mRNA).

### Serum analyses

Blood was collected from the mice via cardiac puncture, serum removed and stored at −80°C. Protease inhibitor was added to sera (Protease Inhibitor Cocktail, VWR Life Science Amresco) and analyzed for metabolic hormones (Mouse Metabolic Array) and a panel of 31 chemokines/cytokines (Mouse Cytokine Array/Chemokine Array 31-Plex) by addressable laser bead immunoassay by Eve Technologies (evetechnologies.com; Calgary, AB, Canada).

### Flow cytometry

#### Isolation of intestinal lamina propria cells

Intestinal lamina propria cells from mice were isolated using a modified version of a previously described protocol.^59^ In brief, colons were removed and placed in cold calcium- and magnesium-free Hanks balanced salt solution (HBSS; Gibco) supplemented with 2% heat-inactivated FCS and 15 mM HEPES (Gibco). Intestines were cut open longitudinally, washed thoroughly, cut into 2 cm pieces, and incubated with shaking in EDTA buffer (HBSS supplemented with 2% FCS, 15 mM HEPES, and 5 mM EDTA) for 60 minutes at 37°C to remove epithelial cells. After removing the supernatant, tissue pieces were incubated in RPMI-1640 (Sigma) supplemented with 10% FCS, 15 mM HEPES, 100 μg/ml DNase I (Roche) and 200 μg/ml collagenase type IV (Sigma) for 40 minutes at 37°C. The cell suspension was filtered through a 70 μm cell strainer (Sigma), washed, and resuspended in FACS buffer (1X PBS supplemented with 2% FBS and 0.5 M NA2EDTA) before proceeding with antibody staining.

For flow cytometry, the cells were incubated with 1 mg/ml rat anti-mouse CD16/CD32 Ab (Fc-block; clone 2.4G2) for 15 min at 4°C and then washed with cold FACS buffer. Fluorochrome-labeled extracellular antibodies were added in a total volume of 100 μl to 1 × 10^6^ cells, mixed thoroughly, and incubated for 25 minutes at 4°C. Extracellular antibodies used in this study are listed below in different panels and were used at a dilution of 1:200. Following extracellular staining, cells were washed with PBS and resuspended in viability dye (Life Technologies) for 20 minutes at 4°C. Intracellular staining for cytokines IFNγ, IL-17, IL-22 and TNF-α was performed using eBioscience™ Intracellular Fixation & Permeabilization Buffer Set. Before staining, cells were stimulated with Cell Stimulation Cocktail (plus protein transport inhibitors) (eBioscience) for 3 hours at 37°C. FoxP3 and RORgT staining was performed using eBioscience™ Foxp3/Transcription Factor Staining Buffer Set, and antibodies were used at a dilution of 1:100. A FACSCanto II (BD Biosciences) was used for sample analysis, and flow cytometric analysis was performed using FlowJo software (TreeStar, Inc). The gating strategy is shown in Figure S4.

### Short-chain fatty acids (SCFAs) analysis

Direct-injection gas chromatography was used to quantify SCFAs acetic, propionic, butyric, valeric, iso-butyric, and iso-valeric acid from cecal samples collected from Muc2^−/−^ mice.^18^ Briefly, cecal samples were homogenized in isopropyl alcohol, containing 2-ethyl butyric acid at 0.01% v/v used as an internal standard and then centrifuged and the supernatant removed. The supernatant was injected into a Trace 1300 Gas Chromatograph, equipped with a flame-ionization detector, with AI1310 autosampler (Thermo Scientific, Walkham, MA, USA) in splitless mode. A fused silica FAMEWAX (Restekas, Bellefonte, PA, USA) column 30 m × 0.32 mm i.d. coated with 0.25 μm film thickness was used. Helium was supplied as the carrier gas at a flow rate of 1.8 ml/min. The initial oven temperature was 80°C, maintained for 5 min, raised to 90°Cat 5°C/min, then increased to 105°C at 0.9°C/min, and finally increased to 240°C at 20°C/min and held for 5 min. The temperature of the flame-ionization detector and the injection port was 240°C and 230°C,respectively. The flow rates of hydrogen, air and nitrogen as makeup gas were 30, 300 and 20 ml/ min, respectively. Data were analyzed with Chromeleon 7 software (Bannockburn, IL, USA). Fine separation of SCFA was confirmed by the complete separation of the volatile-free acid mix (Sigma, Oakville, ON, Canada). Data are presented as absolute values (g of SCFA per g of feces).

### FITC-dextran assessment of intestinal permeability

Mice were gavaged with FITC-dextran (molecular mass, 4 kDa; FD4; Sigma-Aldrich) at a concentration of 80 mg/100 g body weight. Four hours after gavage, blood was collected by cardiac puncture, placed in 3% acid-citrate dextrose, and centrifuged at 1000 xg for 12 minutes to remove serum. Fluorescence of FITC-dextran in serum was diluted to 1:10 with PBS and measured on a Promega GloMax Multi Detection System (Promega) at 490 nm excitation and 520 nm emission wavelengths. FITC-dextran concentration was determined from a standard curve generated by serial dilutions of FITC-dextran.

### Intestinal Alkaline Phosphatase Assay (IAP)

IAP was extracted from 25 mg colon tissues in 500 ul of RIPA buffer (50 mM Tris, pH 8.0, 1% Triton-X 100, 0.5% sodium deoxycholate, 0.1% sodium dodecyl sulfate, 150 mM sodium chloride) and protease inhibitor. The sample was homogenized at 30 Hz for 2 minutes, then centrifuged at 1610 × g for 5 min, and the supernatant containing IAP was collected. The IAP assay was completed using the Alkaline Phosphatase Activity Fluorometric Assay Kit (BioVision) according to the manufacturer’s instructions. Briefly, each 10 ul of each sample was treated with 100 ul of ALP Assay Buffer and 20 ul of 0.5 mM Methylumbelliferyl phosphate disodium salt (MUP) and incubated at 25°C for 30 minutes. Next, 20 ul of an inhibitor (aqueous K2HPO4) was added to each well. In addition, a standard curve was created using a stock solution of 1 mg/mL BSA (Sigma) in triplicates with multiple concentrations. Fluorescence intensity was measured using the Promega GloMax Multi Detection System (Promega) at 360 nm excitation and 440 nm emission. Total relative protein concentration was quantified using the Bio-Rad Protein Assay (Bio-Rad, Ontario, Canada). IAP values are expressed as units of IAP/mg of protein.

### Glucose tolerance test (IPGTT)

At nine weeks, an intraperitoneal glucose tolerance testing (IPGTT) was completed, using standard protocols from the D.I.A.B.E.T.E.S. Center (UBC-Okanagan, Kelowna, BC, Canada). Briefly, a dose of 1 g/kg body weight glucose (20% wt/vol glucose solution) was administered via intraperitoneal injection. Blood sampling and glucose testing was performed at 0, 15, 30, 60, 90 and 120-minutes following glucose injection.

### Microbiome processing

Microbiome analysis was completed on the distal colon samples. The DNA was extracted using the QIAamp PowerFecal Pro DNA kit (Qiagen; Cat No 51804) with an additional wash step to increase DNA purity. Extracted DNA was normalized using a Nanodrop 1000 spectrophotometer, and the V4-V5 region of the 16S ribosomal DNA was amplified using 515 FB and 926 R primers attached to the Illumina adapter overhang. Samples were sequenced by the Integrated Microbiome Resource (Dalhousie University, Halifax, Nova Scotia, Canada) according to their protocol.^60^

### Bioinformatic analysis

Post-sequencing analyses were performed using the QIIME 2 platform (version 2021.4).^61^ Demultiplexed reads from two MiSeq runs were imported into the QIIME 2 environment, and primers were removed using the q2-cutadapt plugin.^62^ Quality control entailed filtering, dereplication, chimera removal, denoising, and merging of paired-end reads on each run separately using the DADA2 plugin with default settings.^63^ The resulting amplicon sequence variant (ASV) tables were merged for downstream analysis. A phylogenetic tree was constructed using a SATé-enabled phylogenetic placement (SEPP) technique as implemented in the q2-fragment-insertion plugin^64^ using a backbone tree built from the Greengenes reference database (version 13.8).^65^ For taxonomic classification, we trained a classifier on the entire length 16S region and incorporated environment-specific abundances weights specific to animal distal gut environment acquired from the *readytowear* repository (https://github.com/BenKaehler/readytowear). This weighted bespoke approach for taxonomic classification has been shown to significantly improve accuracy over common Naive Bayes classification methods.^66^ Before diversity analysis, all ASVs that were not classified at least at the phylum level were discarded as contaminants, and only samples with at least 1,000 sequences were kept. A final table consisting of 7 CO, 8 MD, 9 MF, and 14 OO samples were retained for downstream analysis. Alpha-diversity metrics (Shannon’s diversity index, Faith’s phylogenetic diversity, ASV richness and Pielou’s Evenness) were used.^61^ For beta diversity, we used the q2-DEICODE plug-in^60^ which calculates a form of Aitchison distances that is robust to high levels of sparsity, is compositionally aware, and circumvents the need for rarefying. Using the *q2-beta group significance* plugin, a permutational multivariate analysis of variance (PERMANOVA) test (α = 0.05, with 999 permutations) was run on the robust Aitchison distances to determine differences between diet groups. The PERMANOVA test’s assumption of multivariate dispersion was assessed using a PERMDISP test. We utilized multinomial regression using Songbird^67^ to obtain ASV rankings most associated with each group. These differential ranks were visualized using Qurro ^68^ and the differentials for the top 10 ASVs associated with each group were exported into R^69^ using the *qiime2R* package [https://github.com/jbisanz/qiime2R] for further custom visualization and statistical analysis.

### Statistical analysis

Statistical analyses were performed using R statistical software^69^and GraphPad Prism 9 (GraphPad Software, San Diego, California USA, www.graphpad.com) with *P* values below 0.05 considered statistically significant. The results are expressed as the mean value with a standard error of the mean (SEM). When comparing diet groups, the Kruskal–Wallis test with the Dunn post hoc test was performed for nonparametric data, unless otherwise indicated. For bacterial differential abundance analysis, the log ratio of the top 5 ASVs associated with each diet group to the top 5 ASV associated with MD was calculated and compared across groups using an ANOVA test. The false discovery rate resulting from multiple testing was controlled using the Benjamini-Hochberg method. The violin boxplots with jitters were produced using the ggplot2 (https://cran.r-project.org/web/packages/ggplot2/citation.html) package and further augmented with the ggpubr (https://rpkgs.datanovia.com/ggpubr/) package (Figure S7).

## Supplementary Material

Supplemental Material

## Figures and Tables

**Table:** 

IBD	inflammatory bowel disease
CD	Crohn’s disease
CO	corn oil
IAP	intestinal alkaline phosphatase
MD	Mediterranean diet
MF	milk fat
Muc2^−/−^	mucin 2 knock-out
MUFA	monounsaturated fatty acids
n-3 PUFA	omega-3 polyunsaturated fatty acids
n-6 PUFA	omega-6 polyunsaturated fatty acids
OO	olive oil
SCFA	short chain fatty acids
SFA	saturated fatty acids
UC	ulcerative colitis
WD	Western diet

## Preprint

A Mediterranean-like fat blend protects against the development of severe colitis in the mucin-2 deficient murine model. Natasha Haskey, Jiayu Ye, Mehrbod Estaki, Andrea A. Verdugo Meza, Jacqueline A.Barnett, Mitra Yousefi, Blake W. Birnie, Samantha Gruenheid, Sanjoy Ghosh, Deanna L.Gibson bioRxiv 2021.09.27.462042; doi: https://doi.org/10.1101/2021.09.27.462042

## Synopsis

Various types of dietary fat, independent of total fat content, uniquely influence intestinal inflammation, metabolism, and host-microbe dynamics. We demonstrate in a murine model of spontaneous colitis (Muc2^−/−^ mice) that the fat blend found in the MD (high monounsaturated, 2:1 n-6:n-3 polyunsaturated and moderate saturated fat) contributes to an increased abundance of health-promoting microbes, reduces disease activity, reduces severe colitis, and as well as improves metabolic parameters. The Mediterranean dietary lipid blend can mitigate the damaging effects of chronic inflammation and metabolic abnormalities seen in experimental colitis, therefore could be considered as a maintenance diet for ulcerative colitis.

## References

[1] Verdugo-Meza A, Ye J, Dadlani H, Ghosh S, Gibson DL. Connecting the dots between inflammatory bowel disease and metabolic syndrome: a focus on gut-derived metabolites. Nutrients. 2020.12(5).Published online 2020 doi:10.3390/nu12051434. PMC7285036 32429195

[2] Ramos GP, Papadakis KA . Mechanisms of disease: inflammatory bowel diseases. *Mayo Clinic Proceedings*. 2019 Jan;94(1):155–23. doi:10.1016/j.mayocp.2018.09.013. PMID: 30611442; PMCID: PMC6386158. PMC6386158 30611442

[3] Ng SC, Shi HY, Hamidi N, Underwood FE, Tang W, Benchimol EI, Panaccione R, Ghosh S, Wu JCY, Chan FKL, et al. Worldwide incidence and prevalence of inflammatory bowel disease in the 21st century: a systematic review of population-based studies. The Lancet. 2017.390(10114):2769–2778. Published online 2017. doi:10.1016/S0140-6736(17)32448-0. *The Lancet*. 29050646

[4] Li T, Qiu Y, Yang HS , et al. Systematic review and meta-analysis: Association of a pre-illness Western dietary pattern with the risk of developing inflammatory bowel disease J Dig Dis. 2022 Jul; 21(7): 362–371 . doi:10.1111/1751-2980.12910. PMID: 32463159. 32463159

[5] Christ A, Lauterbach M, Latz E. Western diet and the immune system: an inflammatory connection. Immunity. 2019;51(5):794–811. doi:10.1016/j.immuni.2019.09.020. 31747581

[6] Liu S, Zhao W, Lan P, Mou X. The microbiome in inflammatory bowel diseases: from pathogenesis to therapy. Protein Cell. 2021;12(5):331–345. doi:10.1007/s13238-020-00745-3. 32601832 PMC8106558

[7] Fyderek K, Strus M, Kowalska-Duplaga K, Gosiewski T, Wędrychowicz A, Jedynak-Wąsowicz U, Sładek M, Pieczarkowski S, Adamski P, Kochan P, et al. Mucosal bacterial microflora and mucus layer thickness in adolescents with inflammatory bowel disease. World Journal of Gastroenterology. 2009.15(42):5287–5294. doi:10.3748/wjg.15.5287. 19908336 PMC2776855

[8] Balzola F, Cullen G, Hoentjen F, Ho GT, Russell R. Bacteria penetrate the normally impenetrable inner colon mucus layer in both murine colitis models and patients with ulcerative colitis. Inflammatory Bowel Disease Monitor. 2013;13(4):156–157. doi:10.1136/gutjnl-2012-303207.

[9] van der Sluis M, de Koning Baee, de Bruijn Acjmjm, Van der Sluis M, De Koning BAE, De Bruijn ACJM, Velcich A, Meijerink JPP, Van Goudoever JB, Büller HA, et al. Muc2-Deficient mice spontaneously develop colitis, indicating that muc2 is critical for colonic protection. Gastroenterology. 2006.131(1):117–129. doi:10.1053/j.gastro.2006.04.020. 16831596

[10] Lu P, Burger-Van Paassen N, van der Sluis M , et al. Colonic gene expression patterns of mucin muc2 knockout mice reveal various phases in colitis development. Inflamm Bowel Dis. 2011 Oct;17(10):2047–57. doi:10.1002/ibd.21592.Epub 2011 Jan 6. PMID: 21910166. 21910166

[11] Wu M, Wu Y, Li J, Bao Y, Guo Y, Yang W . The Dynamic Changes of Gut Microbiota in Muc2 Deficient Mice. Int J Mol Sci. 2018 Sep 18;19(9):2809. doi:10.3390/ijms19092809.PMID: 30231491; PMCID: PMC6164417. PMC6164417 30231491

[12] Morampudi V, Dalwadi U, Bhinder G , et al. The goblet cell-derived mediator RELM-β drives spontaneous colitis in Muc2-deficient mice by promoting commensal microbial dysbiosis. Mucosal Immunol. 2016 Sep;9(5):1218–1233. doi:10.1038/mi.2015.140. Epub 2016 Jan 27. PMID: 26813339. 26813339

[13] IBD in EPIC Study Investigators, Tjonneland A, Overvad K, Bergmann MM, Nagel, G, Linseisen, J, Hallmans, G, Palmqvist, R, Sjodin, H, Hagglund, G, Berglund, G et al , et al. Linoleic acid, a dietary n-6 polyunsaturated fatty acid, and the aetiology of ulcerative colitis: a nested case-control study within a European prospective cohort study. Gut. 2009 Dec;58(12):1606–1611. doi:10.1136/gut.2008.169078. 19628674

[14] Ananthakrishnan AN, Khalili H, Konijeti GG , et al. Long-term intake of dietary fat and risk of ulcerative colitis and Crohn’s disease. Gut. 2014 May.63(5):776–784. doi:10.1136/gutjnl-2013-305304. Epub 2013 Jul 4. PMID: 23828881; PMCID: PMC3915038. 23828881 PMC3915038

[15] Sasson AN, Ingram RJM, Zhang Z , et al. The role of precision nutrition in the modulation of microbial composition and function in people with inflammatory bowel disease. The Lancet Gastroenterology & Hepatology. 2021 Sep;6(9):754–769. doi:10.1016/S2468-1253(21)00097-2. Epub 2021 Jul 14. PMID: 34270915. 34270915

[16] Abulizi N, Quin C, Brown K, Chan YK, Gill SK, Gibson DL. Gut mucosal proteins and bacteriome are shaped by the saturation index of dietary lipids. Nutrients. 2019.11(2). doi:10.3390/nu11020418. PMC6412740 30781503

[17] DeCoffe D, Quin C, Gill SK, Tasnim N, Brown K, Godovannyi A, Dai C, Abulizi N, Chan YK, Ghosh S, et al. Dietary lipid type, rather than total number of calories, alters outcomes of enteric infection in mice. Journal of Infectious Diseases. 2016.213(11):1846–1856. doi:10.1093/infdis/jiw084. 27067195

[18] Ye J, Haskey N, Dadlani H , et al. Deletion of mucin 2 induces colitis with concomitant metabolic abnormalities in mice. American Journal of Physiology - Gastrointestinal and Liver Physiology. 2021 May;320(5):G791–G803. doi:10.1152/AJPGI.00277.2020. Epub 2021 Mar 17. PMID: 33728986. 33728986

[19] Bach-Faig A, Berry EM, Lairon D, Reguant J, Trichopoulou A, Dernini S, Medina FX, Battino M, Belahsen R, Miranda G, et al. Mediterranean diet pyramid today. Science and cultural updates. Public Health Nutr. 2011.14(12A):2274–2284. doi:10.1017/S1368980011002515. 22166184

[20] Kim JJ, Shajib MS, Manocha MM, Khan WI . Investigating intestinal inflammation in DSS-induced model of IBD. Journal of Visualized Experiments. 2012 Feb 1;(60): 3678. doi:10.3791/3678. PMID: 22331082; PMCID: PMC3369627. 22331082 PMC3369627

[21] Nunes T, Bernardazzi C, de Souza HS. Cell death and inflammatory bowel diseases: apoptosis, necrosis, and autophagy in the intestinal epithelium. Biomed Res Int. 2014.2014:1–12. doi:10.1155/2014/218493. PMC4121991 25126549

[22] Edelblum KL, Yan F, Yamaoka T, Polk DB. Regulation of apoptosis during homeostasis and disease in the intestinal epithelium. Inflamm Bowel Dis. 2006;12(5):413–424. doi:10.1097/01.MIB.0000217334.30689.3e. 16670531

[23] Williams IR, Parkos CA. Colonic neutrophils in inflammatory bowel disease: double-edged swords of the innate immune system with protective and destructive capacity. Gastroenterology. 2007;133(6):2049–2052. doi:10.1053/j.gastro.2007.10.031. 18054577

[24] Imam T, Park S, Kaplan MH, Olson MR. Effector T helper cell subsets in inflammatory bowel diseases. Front Immunol. 2018.9 Published online 2018. doi:10.3389/fimmu.2018.01212. PMC5992276 29910812

[25] Al-Haddad S, Riddell RH, Riddell RH. The role of eosinophils in inflammatory bowel disease. Gut. 2005;54(12):1674–1675. doi:10.1136/gut.2005.072595. 16284283 PMC1774805

[26] Neurath MF . Cytokines in inflammatory bowel disease. Nat Rev Immunol. 2014 May;14(5):329–42. doi:10.1038/nri3661. Epub 2014 Apr 22. PMID: 24751956. 24751956

[27] Stagg AJ, Li P, Fan L, Wu M. Intestinal dendritic cells in health and gut inflammation. Front Immunol. 2018.9:9. doi:10.3389/fimmu.2018.02883. 30574151 PMC6291504

[28] Gálvez J. Role of Th17 cells in the pathogenesis of human IBD. ISRN Inflammation. 2014.2014:1–14. doi:10.1155/2014/928461. PMC4005031 25101191

[29] Lindemans CA, Calafiore M, Mertelsmann AM, O’Connor MH, Dudakov JA, Jenq RR, Velardi E, Young LF, Smith OM, Lawrence G, et al. Interleukin-22 promotes intestinal-stem-cell-mediated epithelial regeneration. Nature. 2015.528:7583. doi:10.1038/nature16460. PMC4720437 26649819

[30] Cranford TL, Enos RT, Velázquez KT, McClellan JL, Davis JM, Singh UP, Nagarkatti M, Nagarkatti PS, Robinson CM, Murphy EA, et al. Role of MCP-1 on inflammatory processes and metabolic dysfunction following high-fat feedings in the FVB/N strain. Int J Obes. 2016.40(5):844–851. doi:10.1038/ijo.2015.244. PMC4854829 26620890

[31] Meshkibaf S, Martins AJ, Henry GT, Kim SO, Chu C-Q. Protective role of G-CSF in dextran sulfate sodium-induced acute colitis through generating gut-homing macrophages. Cytokine. 2016.101:78. doi:10.1016/j.cyto.2015.11.025. 26687628

[32] Couper KN, Blount DG, Riley EM . IL-10: the master regulator of immunity to infection The Journal of Immunology. 2008 May 1;180(9): 5771–7. doi:10.4049/jimmunol.180.9.5771. PMID: 18424693. 18424693

[33] Paz-Filho G, Wong ML, Licinio J, Mastronardi C. Leptin therapy, insulin sensitivity, and glucose homeostasis. Indian J Endocrinol Metab. 2012;16(9):549. doi:10.4103/2230-8210.105571. PMC3602983 23565489

[34] Lallès JP. Intestinal alkaline phosphatase: multiple biological roles in maintenance of intestinal homeostasis and modulation by diet. Nutrition Reviews. 2010.68(6):323–332. Published online 2010. doi:10.1111/j.1753-4887.2010.00292.x. *Nutrition Reviews*. 20536777

[35] Parada Venegas D, la Fuente Mk D, Landskron G , et al. Short chain fatty acids (SCFAs)-Mediated gut epithelial and immune regulation and its relevance for inflammatory bowel diseases. Front Immunol. 2019 Mar.11;10:277. doi:10.3389/fimmu.2019.00277. Erratum in: Front Immunol. 2019 Jun 28;10:1486. PMID: 30915065; PMCID: PMC6421268. 30915065 PMC6421268

[36] Laffin M, Fedorak R, Zalasky A , et al. A high-sugar diet rapidly enhances susceptibility to colitis via depletion of luminal short-chain fatty acids in mice. Sci Rep. 2019 Aug;9(1):12294. doi:10.1038/s41598-019-48749-2. PMID: 31444382; PMCID: PMC6707253. 31444382 PMC6707253

[37] Heinsen FA, Knecht H, Neulinger SC , et al. Dynamic changes of the luminal and mucosaassociated gut microbiota during and after antibiotic therapy with paromomycin. Gut Microbes. 2015 Jul 4;6(4): 243–54. doi:10.1080/19490976.2015.1062959. PMID: 26178862; PMCID: PMC4615565. 26178862 PMC4615565

[38] Ramasamy S, Nguyen DD, Eston MA, Nasrin Alam S, Moss AK, Ebrahimi F, Biswas B, Mostafa G, Chen KT, Kaliannan K, et al. Intestinal alkaline phosphatase has beneficial effects in mouse models of chronic colitis. Inflamm Bowel Dis. 2011.17(2):532–542. doi:10.1002/ibd.21377. 20645323 PMC3154118

[39] Hildebrandt MA, Hoffmann C, Sherrill-Mix SA, Keilbaugh SA, Hamady M, Chen Y, Knight R, Ahima RS, Bushman F, Wu GD, et al. High-Fat diet determines the composition of the murine gut microbiome independently of obesity. Gastroenterology. 2009.137(5):1716–1724.e2. doi:10.1053/j.gastro.2009.08.042. 19706296 PMC2770164

[40] Kushiyama A, Shojima N, Ogihara T, Inukai K, Sakoda H, Fujishiro M, Fukushima Y, Anai M, Ono H, Horike N, et al. Resistin-like molecule β activates MAPKs, suppresses insulin signaling in hepatocytes, and induces diabetes, hyperlipidemia, and fatty liver in transgenic mice on a high fat diet. J of Biol Chem. 2005.280(51):42016–42025. doi:10.1074/jbc.M503065200. 16243841

[41] Ikemoto S, Takahashi M, Tsunoda N, Maruyama K, Itakura H, Ezaki O. High-fat diet-induced hyperglycemia and obesity in mice: differential effects of dietary oils. Metabolism. 1996;45(12):1539–1546. doi:10.1016/S0026-0495(96)90185-7. 8969289

[42] Rabiee A, Galiatsatos P, Salas-Carrillo R, Thompson MJ, Andersen DK, Elahi D. Pancreatic polypeptide administration enhances insulin sensitivity and reduces the insnlin requirement of patients on insulin pump therapy. J Diabetes Sci Technol. 2011;5(6):1521–1528. doi:10.1177/193229681100500629. 22226275 PMC3262724

[43] Li X, Wei X, Sun Y , et al. High-fat diet promotes experimental colitis by inducing oxidative stress in the colon. American Journal of Physiology - Gastrointestinal and Liver Physiology. 2019 Oct 1;317(4):G453–G462. doi:10.1152/ajpgi.00103.2019. Epub 2019 Aug 14. PMID: 31411504. 31411504

[44] Hibberd AA, Lyra A, Ouwehand AC , et al. Intestinal microbiota is altered in patients with colon cancer and modified by probiotic intervention. BMJ Open Gastroenterology. 2017 Jul 3;4(1):e000145. doi:10.1136/bmjgast-2017-000145. PMID: 28944067; PMCID: PMC5609083. PMC5609083 28944067

[45] Butera A, Di Paola M, Pavarini L , et al. Nod2 deficiency in mice is associated with microbiota variation favouring the expansion of mucosal cd4+ lap+ regulatory cells. Sci Rep. 2018 Sep 24;8(1):14241. doi:10.1038/s41598-018-32583-z. PMID: 30250234; PMCID: PMC6155205. 30250234 PMC6155205

[46] Sonnenburg JL, Sonnenburg ED. Vulnerability of the industrialized microbiota. Science. 2019;366(6464):6464. doi:10.1126/science.aaw9255. 31649168

[47] Feng Q, Liang S, Jia H , et al. Gut microbiome development along the colorectal adenoma-carcinoma sequence. Nat Commun. 2015 Mar 11;6:6528. doi:10.1038/ncomms7528. PMID: 25758642. 25758642

[48] Berry D, Schwab C, Milinovich G , et al. Phylotype-level 16S rRNA analysis reveals new bacterial indicators of health state in acute murine colitis. ISME Journal. 2012 Nov;6(11):2091–2106. doi:10.1038/ismej.2012.39. Epub 2012 May 10. PMID: 22572638; PMCID: PMC3475367. PMC3475367 22572638

[49] Ganesh BP, Klopfleisch R, Loh G, Blaut M. Commensal akkermansia muciniphila exacerbates gut inflammation in salmonella Typhimurium-Infected gnotobiotic mice. PLoS ONE. 2013;8(9):e74963. doi:10.1371/journal.pone.0074963. 24040367 PMC3769299

[50] Crost EH, Tailford LE, Le Gall G, Fons M, Henrissat B, Juge N. Utilisation of mucin glycans by the human gut symbiont ruminococcus gnavus is Strain-Dependent. PLoS ONE. 2013;8(10):e76341. doi:10.1371/journal.pone.0076341. 24204617 PMC3808388

[51] Bloom SM, Bijanki VN, Nava GM , et al. Commensal Bacteroides species induce colitis in host-genotype-specific fashion in a mouse model of inflammatory bowel disease. Cell Host Microbe. 2011 May 19;9(5):390–403. doi:10.1016/j.chom.2011.04.009. PMID: 21575910; PMCID: PMC3241010. 21575910 PMC3241010

[52] Berry D, Kuzyk O, Rauch I , et al. Intestinal microbiota signatures associated with inflammation history in mice experiencing recurring colitis Front Microbiol. 2015 Dec 15;6:1408. doi:10.3389/fmicb.2015.01408. PMID: 26697002; PMCID: PMC4678223. 26697002 PMC4678223

[53] Stidham RW, Higgins PDR. Colorectal cancer in inflammatory bowel disease. Clin Colon Rectal Surg. 2018;31(3):168–178. doi:10.1055/s-0037-1602237. 29720903 PMC5929884

[54] Herp S, Brugiroux S, Garzetti D, Ring D, Jochum LM, Beutler M, Eberl C, Hussain S, Walter S, Gerlach RG, et al. Mucispirillum schaedleri antagonizes salmonella virulence to protect mice against colitis. Cell Host Microbe. 2019.25(5):681–694.e8. doi:10.1016/j.chom.2019.03.004. 31006637

[55] Fukuda S, Toh H, Hase K , et al. Bifidobacteria can protect from enteropathogenic infection through production of acetate. Nature. 2011 Jan 27;469(7331):543–7. doi:10.1038/nature09646. PMID: 21270894. 21270894

[56] Tedelind S, Westberg F, Kjerrulf M, Vidal A. Anti-inflammatory properties of the short-chain fatty acids acetate and propionate: a study with relevance to inflammatory bowel disease. World Journal of Gastroenterology. 2007;13(20):2826. doi:10.3748/wjg.v13.i20.2826. 17569118 PMC4395634

[57] Cooper HS, Murthy SNS, Shah RS, Sedergran DJ. Clinicopathologic study of dextran sulfate sodium experimental murine colitis. Laboratory Investigation. Published online 1993. doi:10.1016/S0021-5198(19)41298-5. 8350599

[58] Bergstrom KSBB, Kissoon-Singh V, Gibson DLet al, Muc2 protects against lethal infectious colitis by disassociating pathogenic and commensal bacteria from the colonic mucosa. PLoS Pathogens. 2010;6(5):e1000902. doi:10.1371/journal.ppat.1000902 20485566 PMC2869315

[59] Weigmann B, Tubbe I, Seidel D, Nicolaev A, Becker C, Neurath MF. Isolation and subsequent analysis of murine lamina propria mononuclear cells from colonic tissue. Nature Protocols. 2007;2(10):2307–2311. doi:10.1038/nprot.2007.315. 17947970

[60] Comeau AM, Douglas GM, Langille MG. Microbiome Helper: a Custom and Streamlined Workflow for Microbiome Research. mSystems. 2017 Jan 3;2(1)e00127-16. doi: 10.1128/mSystems.00127-16. PMC5209531 28066818

[61] Bolyen E, Rideout JR, Dillon MR, Bokulich NA, Abnet CC, Al-Ghalith GA, Alexander H, Alm EJ, Arumugam M, Asnicar F, et al. Reproducible, interactive, scalable and extensible microbiome data science using QIIME 2. Nat Biotechnol. 2019.37(8):852–857. doi:10.1038/s41587-019-0209-9. 31341288 PMC7015180

[62] Martin M. Cutadapt removes adapter sequences from high-throughput sequencing reads. EMBnet.journal. 2011.17(1). doi:10.14806/ej.17.1.200.

[63] Callahan BJ, McMurdie PJ, Rosen MJ, Han AW, Johnson AJA, Holmes SP. DADA2: high-resolution sample inference from Illumina amplicon data. Nat Methods. 2016.13(7):581–3. Published online 2016. doi:10.1038/nature09646. 27214047 PMC4927377

[64] Janssen S, McDonald D, Gonzalez A , et al. Phylogenetic placement of exact amplicon sequences improves associations with clinical information. mSystems. 2018 Apr 17;3(3):e00021–18. doi:10.1128/msystems.00021-18. PMID: 29719869; PMCID: PMC5904434. 29719869 PMC5904434

[65] McDonald D, Price MN, Goodrich J , et al. An improved Greengenes taxonomy with explicit ranks for ecological and evolutionary analyses of bacteria and archaea. ISME Journal. 2012 Mar;6(3):610–618. doi:10.1038/ismej.2011.139. Epub 2011 Dec 1. PMID: 22134646; PMCID: PMC3280142. PMC3280142 22134646

[66] Kaehler BD, Bokulich NA, McDonald D, Knight R, Caporaso JG, Huttley GA. Species abundance information improves sequence taxonomy classification accuracy. Nat Commun. 2019.10(1). doi:10.1038/s41467-019-12669-6. PMC6789115 31604942

[67] Morton JT, Marotz C, Washburne A , et al. Establishing microbial composition measurement standards with reference frames. Nat Commun. 2019 Jun 20;10(1):2719. doi:10.1038/s41467-019-10656-5. PMID: 31222023; PMCID: PMC6586903. 31222023 PMC6586903

[68] Fedarko MW, Martino C, Morton JT, González A, Rahman G, Marotz CA, Minich JJ, Allen EE, Knight R. Visualizing 'omic feature rankings and log-ratios using Qurro. NAR Genom Bioinform. 2020 Jun;2(2):lqaa023. doi: 10.1093/nargab/lqaa023. Epub 2020 Apr 28. PMID: 32391521; PMCID: PMC7194218. 32391521 PMC7194218

[69] R Core Team (2020). R: A language and environment for statistical computing. R Foundation for Statistical Computing, Vienna, Austria. https://www.R-project.org/.

